# Fanconi-Bickel syndrome in two Palestinian children: marked phenotypic variability with identical mutation

**DOI:** 10.1186/s13104-016-2184-2

**Published:** 2016-08-04

**Authors:** Imad Mohammad Dweikat, Issa Shaher Alawneh, Sami Fares Bahar, Mutaz Idrees Sultan

**Affiliations:** 1 Pediatric Department, Faculty of Medicine and Health Sciences, An-Najah National University, P.O.Box 7, 44839 Nablus, Palestine Israel; 2 Pediatric Department, Makassed Hospital, Mount of Olives, P.O.Box 19482, Jerusalem, Palestine Israel

**Keywords:** FBS, Hepatomegaly, Rickets, Short stature, Hypoglycemia

## Abstract

**Background:**

Fanconi-Bickel syndrome (FBS, OMIM 227810) is a rare autosomal recessive disease caused by a deficiency of glucose transporter 2 (GLUT2), a member of the facilitative glucose transporter family (Santer et al. J Inherit Metab Dis 21:191–194, 1998). The typical clinical picture is characterized by hepatorenal glycogen accumulation resulting in hepato- and nephromegaly, impaired utilization of glucose and galactose, proximal renal tubular dysfunction, rickets and severe short stature.

**Case presentation:**

We report 2 Palestinian patients from 2 families who were homozygous for the mutation p.R301X (C>T) in exon 7of *GLUT2* gene. Patient 1 showed clinical and laboratory improvement with age characterized by normal growth and resolution of rickets. Patient 2 had severe phenotype characterized by progressive weight loss, persistent metabolic acidosis, marked polyuria and clinical and laboratory findings of rickets progressing to death at age 10 months.

**Conclusion:**

This report further expands the clinical spectrum of FBS even with identical mutations. Other yet unknown genetic, environmental or stochastic factors may be responsible for phenotypic variability

## Background

A functional loss of GLUT2 is compatible with the clinical symptoms observed in FBS patients [1]. GLUT2 is expressed in hepatocytes, enterocytes and pancreatic β-cell membranes and is involved in the transcellular monosaccharide transport of renal tubular cells and enterocytes [1, 2]. The main features of this disorder are hepatorenal glycogen accumulation, proximal renal tubular dysfunction, impaired utilization of glucose and galactose, rickets and severe short stature [3]. Biochemically, the disease is characterized by a general renal proximal tubular defect (glucosuria, bicarbonate wasting, aminoaciduria, renal tubular acidosis, hyperphosphaturia) and carbohydrate abnormalities that include postprandial hyperglycemia, fasting hypoglycemia and hypergalactosemia [3, 4]. Mutations of *GLUT2* (*SLC2A2*) are the basic defect in FBS patients with characteristic clinical features and also in patients with atypical clinical signs such as intestinal malabsorption, failure to thrive, the absence of hepatomegaly or renal hyperfiltration [5]. These mutations are scattered over the whole coding sequence of the *SLC2A2* gene and none of these mutations is particularly frequent which makes molecular genetic diagnosis laborious [5, 6]. FBS is generally considered a well-defined clinical condition and the overall prognosis seems to be favorable [2, 7]. Phenotypic variability in FBS has been reported and includes atypical phenotype, unusually mild clinical course, severe phenotype and also variable clinical severity in patients with identical mutations [2–8].

We report the first 2 Palestinian patients with FBS from 2 different families who are unrelated and from different geographic location. They are homozygous for the mutation p.R301X in exon 7 of the *GLUT2* gene. Both presented with the typical phenotype of FBS but showed marked variation in the clinical progression of the disease.

## Case presentation

### Case 1

The 6 year old female was born at term to consanguineous parents after uneventful pregnancy. Birth weight was 2800 g (−1.2 SD), length and occipitofrontal circumference at birth were not recorded. Other 3 male siblings are healthy with no symptoms suggestive of FBS and no other family member with diabetes mellitus. First hospitalization was at age 17 month for evaluation of inadequate weight gain and abdominal distension. Her weight was 6600 g (−5.3 SD), length 69 cm (−3.3 SD) and occipitofrontal circumference 43 cm (−2.6 SD). Physical examination showed hepatomegaly and doll-like face. Laboratory findings included high serum triglycerides, mildly elevated serum aspartate aminotransferase, alanine aminotransferase, low phosphorous and elevated alkaline phosphatase. She had polyuria >6 ml/kg/h, massive generalized aminoaciduria, phosphaturia and glucosuria. Serum ammonia, lactic acid, calcium, parathyroid hormone and 25-hydroxyvitamin D were normal (Table 1). Abdominal ultrasound revealed hepatomegaly and minimal calcification of both renal calyces. Radiological findings included generalized osteopenia and rachitic changes. Genetic analysis showed that the patient is homozygous for the mutation R301X (C>T) in exon 7 of the *GLUT2* gene. Treatment included anhydrous phosphate (Joulie’s) solution 5 ml given every 4 h, 5 times daily (0.75 g/24 h), alpha D3 drops (alphacalcidol) 1 µg/day, galactose-restricted diet and uncooked cornstarch 1.6 g/kg given every 6 h during night time to prevent hypoglycemia. Metabolic acidosis was mild and did not require treatment.

**Table 1:** Biochemical findings of the two patients during the first and the last hospitalization

Laboratory test	Patient 1		Patient 2		Normal values
	Initial values age 17 months	Last values age 6 year	Initial values age 5 months	Last values age 8 months	
Serum Ca (mg/dl)	9.8	9.8	9.8	9.8	9–11
Serum PO_4_	2.3 mg/dl	3.8 mg/dl	1.3 mg/dl	1.7 mg/dl	1 month–2 year: 3.8–6.5 g/dl 3–9 year: 3.2–5.8 mg/dl
Serum alkaline phosphatase (U/l)	666	206	1200	1178	150–420
Parathyroid hormone (ng/l)	47	49	13	83	10–65
Vitamin D3 (pg/ml)	25		24	27	16–65
Serum triglyceride (mg/dl)	761	187	1200	254	27–125
Serum cholesterol (mg/dl)	210	186	400	153	<170
Aspartate aminotransferase (U/l)	49	37	140	117	9–80
Alanine aminotransferase (U/l)	76	32	61	56	13–46
Random blood sugar (mg/dl)	120	135	102	88	<126
Serum uric acid (mg/dl)	1.5		1.5	1.1	2.2–6.6
Venous pH	7.32	7.42	7.25	7.30	7.35–7.45
HCO_3_ (mEq/l)	17	22	13	14	18–24
TRP (%)	59	69	32	33	>85
Urine calcium excretion (mg/kg/day)	7.4	5.6	3.2		<4
Glomerular filtration rate (ml/min/1.73 m^2^)	83	226	13	98	49–165
Urine protein excretion (mg/m^2^/h)	64	18.8	42	44	<4
Urine glucose excretion (g/24 h)		25.7	1.8	4.5	

The clinical phenotype was characterized by improvement of growth parameters with age. Rickets resolved with normalization of serum phosphate, alkaline phosphatase and triglyceride. She still shows laboratory findings of renal tubular dysfunction including polyuria, massive aminoaciduria, glucosuria and phosphaturia.

**Fig. 1:**
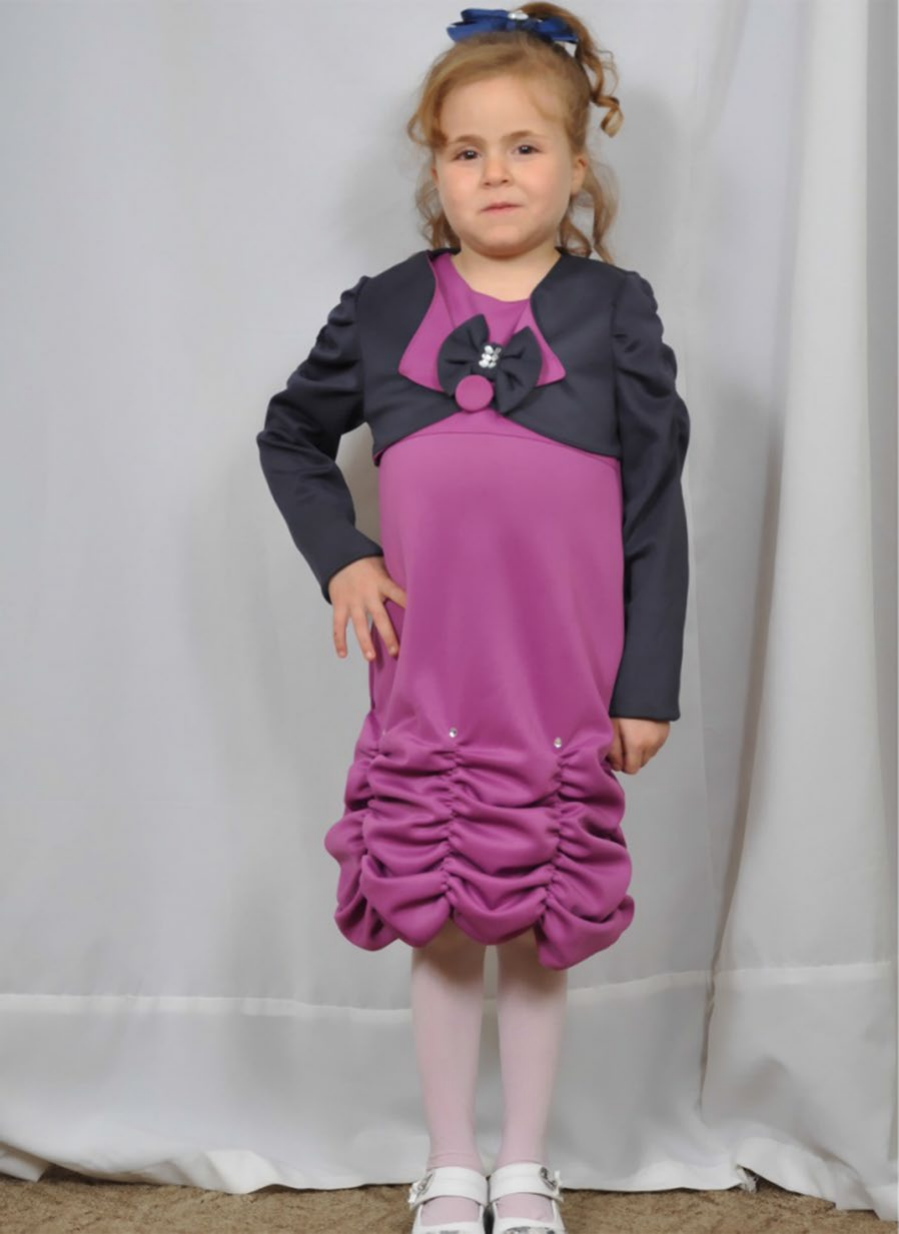
Showing normal growth of case 1 at age 6 years

At age 6 years, her weight was 18 kg (−0.8 SD) and height 107 cm (−1.6 SD) (Fig. 1). Physical exam showed mild hepatomegaly and renal ultrasound showed resolution of calyceal calcifications.

### Case 2

The patient was the first born child to consanguineous parents at term after uneventful pregnancy at 36 weeks gestation. His birth weight was 2200 g (−2 SD). Length and occipitofrontal circumference at birth were not recorded. No other family members with symptoms suggestive of FBS or other family member with diabetes mellitus. At age 2 months, noted to have inadequate weight gain and enlarged anterior fontanel. Different milk formulas were tried but did not result in weight gain. Hospitalized at age 5 months for evaluation of severe failure to thrive, Hyperlipidemia and metabolic acidosis. His weight was 4500 g (−4.3 SD), length 50 cm (−8.8 SD). Physical examination showed abdominal distension, hepatomegaly and doll-like face. He also had rachitic rosaries and wide wrist joints. Laboratory findings included very low serum phosphate, high serum alkaline phosphatase and triglycerides, mildly elevated serum cholesterol, alanine aminotransferase, aspartate aminotransferase and metabolic acidosis. He had massive generalized aminoaciduria, phosphaturia, proteinuria and glucosuria. Serum calcium, parathyroid hormone and 25-hydroxyvitamin D3 were normal as well as urinary excretion of calcium. Other normal laboratory findings included coagulation studies, serum ammonia and lactic acid (Table 1).

Radiological findings showed generalized osteopenia and rachitic changes in the wrist and knee joints. Genetic analysis also showed that the patient is homozygous for the mutation R301X (C>T) in exon 7 of the *GLUT2* gene.

The clinical course was characterized by severe polyuria, inability to gain weight, metabolic acidosis despite treatment with uncooked cornstarch 1.6 g/kg given every 5 h during night time, lactose-free infant formula, oral phosphate supplement at initial dose 0.5 g/24 h given every 4 h, 5 times daily, 1,25-hydroxyvitamin D3 0.5 µg/day and oral sodium bicarbonate supplement 1 mEq/kg given 4 times daily. The medications were given appropriately in terms of both dosage and frequency. Rehospitalized at age 8 months for weight loss, persistent metabolic acidosis and polyuria. Weight was 3.5 kg (−8.9 SD). Indomethacin was added to reduce polyuria but urine output remained persistently elevated >8 ml/kg/h.

The patient died at age 10 months.

## Discussion

The clinical picture of FBS has been thought to be rather homogenous and characterized as a combination of hepatomegaly secondary to glycogen accumulation, galactose intolerance, impaired glucose homeostasis with fasting hypoglycemia and postprandial hyperglycemia, proximal tubular nephropathy and, very typically, severely stunted growth [2, 4, 7].

Our patients presented with the typical phenotype described in the introduction. However, a distinctive feature is the wide variability of clinical course showing normal stature and normal physical exam at age 6 years in patient 1 and a rapidly progressive course characterized by weight loss, massive polyuria, metabolic acidosis and persistence of radiological and biochemical signs of rickets progressing to death in patient 2.

Phenotypic variability has been reported in eight patients from a single Bedouin sibship, all were homozygous for the p.R301X mutation. All had failure to thrive and/or hepatomegaly, fluctuations in blood glucose levels and proximal tubular dysfunction evidenced by massive glucosuria, generalized aminoaciduria, hypercalciuria and hyperphosphaturia. Skeletal involvement ranged from none to radiological and/or clinical signs of rickets and osteopenia. A spectrum of diseases severity was evident during follow up regarding the growth parameters, hospitalizations for disease exacerbations, mean amount of electrolyte replacement therapy, and skeletal and renal complications [4]. 3 patients had mild phenotype; two needed small amounts of replacement therapy and one patient did not require replacement therapy since he was under the authors’ follow up at age 16 years. On the other hand, two patients had severe phenotype characterized by hypocalcemic titanic events in one patient and recurrent symptomatic nocturnal hypoglycemia necessitating gastrostomy for continuous night feedings until age 3 years in the other patient.

Our patients had similar phenotype initially and both had clinical and radiological signs of rickets and osteopenia. The main distinction was the rapidly progressive deterioration in patient 2 in our report despite adequate replacement therapy.

The R301X mutation has also been reported in four unrelated families among 33 different *SLC2A2* mutations in 49 patients with a clinical diagnosis of FBS [5]. No specific case descriptions was provided and the diagnosis was based on the combination of typical clinical and laboratory signs. The authors postulated that it is premature to discuss a genotype–phenotype correlation in FBS and there is no indication that FBS having missense mutations show a milder clinical course when compared with those having truncated mutations.

Unusually mild phenotype has been described in a 9-year old boy characterized by absence of hepato- and nephromegaly, normal growth, normal glucose tolerance even after glucose loading and very mild glucosuria, aminoaciduria and proteinuria [2]. Another mild phenotype was described in a patient with FBS harboring a novel mutation in the GLUT2 gene detected by neonatal screening for galactosemia. He was found to be homozygous for a 3 bp deletion (425-7/delta) within exon 3. Treatment with phosphate and bicarbonate at age 16 months resulted in marked growth improvement and on a Mediterraniean free diet with fractionated meals, he has never shown symptomatic hypoglycemia [8]. Patient 1 in our report has a much similar phenotype showing improvement of growth parameters and absence of carbohydrate abnormalities.

A significant combined defect of the muscle respiratory chain complexes I, III and IV has been described in an 8 year old boy with FBS who had a novel homozygous base exchange at position IVS5+5 of the *GLUT2* gene [7]. The authors hypothesized that FBS patients are prone to a severe secondary respiratory chain defect that could in part explain the heterogeneity of the clinical symptoms. Patient 2 in our report had persistent metabolic acidosis which may be explained by this hypothesis but muscle biopsy for respiratory chain complexes was not performed.

Diabetic ketoacidosis has been described in a female infant with FBS at age 33 days [9]. Although FBS is characterized by carbohydrate abnormalities including posprandial hyperglycemia and fasting hypoglycemia, our patients did not show significant hyperglycemia at presentation or during follow up.

## Conclusion

There is an increasingly recognized evidence that FBS is clinically heterogenous. This report further expands the clinical spectrum of FBS even with identical mutations. Other yet unknown genetic, environmental or stochastic factors may be responsible for phenotypic variability.

## Abbreviations

FBS: Fanconi-Bickel syndrome
GLUT2: glucose transporter 2
FTT: failure to thrive
